# Supplementary material for: Association between oral microbiome and seven types of cancers in East Asian population: a two-sample Mendelian randomization analysis
Source: Front Mol Biosci. 2023 Nov 21;10:1327893. doi: 10.3389/fmolb.2023.1327893 (PMC10702768; doi:10.3389/fmolb.2023.1327893)
Supplement: Supplementary file 1 [file DataSheet1.ZIP › Supplementary Materials/MR plots for tongue/tongue═╝/Gastric cancer/pheno.3124_to_gastric cancer_forest.pdf]

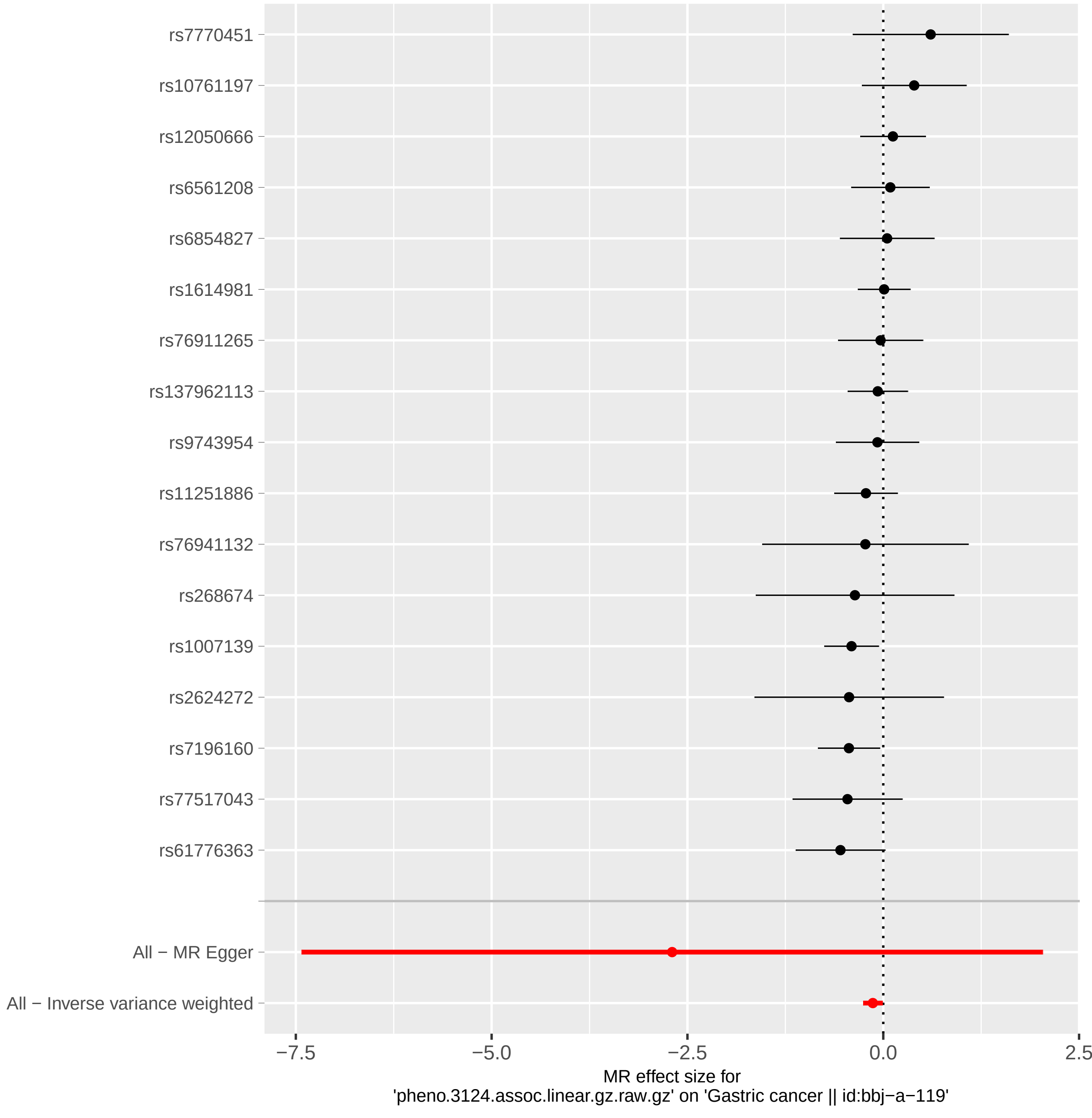
rs7770451
rs10761197
rs12050666
rs6561208
rs6854827
rs1614981
rs76911265
rs137962113
rs9743954
rs11251886
rs76941132
rs268674
rs1007139
rs2624272
rs7196160
rs77517043
rs61776363
All − MR Egger
All − Inverse variance weighted
−7.5
−5.0
−2.5
0.0
2.5
MR effect size for
'pheno.3124.assoc.linear.gz.raw.gz' on 'Gastric cancer || id:bbj−a−119'
